# Transitioning to Minimal Footwear: a Systematic Review of Methods and Future Clinical Recommendations

**DOI:** 10.1186/s40798-017-0096-x

**Published:** 2017-09-15

**Authors:** Joe P. Warne, Allison H. Gruber

**Affiliations:** 1 Department of Applied Science, Institute of Technology Tallaght, Blessington Rd, Dublin 24, Ireland; 2 Setanta College, Thurles Chamber of Enterprise Centre, Thurles, Tipperary Ireland; 3 Department of Kinesiology, Indiana University, Bloomington, IN USA

## Abstract

**Background:**

Recent interest in barefoot running has led to the development of minimalist running shoes that are popular in distance runners. A careful transition to these shoes has been suggested and examined in the literature. However, no guidelines based on systematic evidence have been presented.

The purpose of this review is to systematically examine the methods employed in the literature to transition to minimal footwear (MFW), as well as the outcomes to these studies in distance runners. In addition, MFW transition guidelines for future clinical practice will be presented based on observations from this review.

**Methods:**

A systematic database search was employed using PubMed online as the primary database. Twenty papers were included in the final review.

**Results:**

All studies implemented a prospective transition design to MFW with a detail of this transition provided, which increased MFW exposure up to an average of 60% (30–100%) at completion. Only 8/20 studies included injury prevention exercises, and 9/20 included gait retraining. The main outcomes of this transition included limited positive evidence of transitioning into MFW for running economy (*n* = 4 studies) and muscle development (*n* = 5). The injury incidence comparing running during the MFW transition (17.9 injuries per 100 participants) to matched participants in conventional running shoes (13.4 injuries per 100) appears equivocal (*p* = 0.219; effect size phi (*φ*) = 0.06 [very small]). Finally, several important recommendations for clinical practice and future research have been presented.

**Conclusions:**

It is hoped that this paper will present important first steps in unifying the process of transitioning to MFW, both for academic and clinical use.

## Key Points

- Many minimal footwear (MFW) transition studies have adopted a careful progression of exposure to MFW over time; however, the components of this transition vary considerably.
- The MFW transition literature presents some limited evidence of benefits to running economy, performance, and muscle development.
- Injury incidence comparing transitioning MFW participants to control groups appears equivocal.
- Guidelines and suggestions for a MFW transition have been presented.

## Background

Endurance running remains one of the most popular mass participation sports on the planet. Current figures suggest that over 54 million people currently engage in this sport, as a recreational or competitive activity [1]. However, recent evidence suggests that the rate of injury in runners can vary from 3.2% in cross-country runners to 84.9% in novice runners [2].

With this high risk of injury with running activity, there has been an increase in interest in barefoot running over the last decade [3, 4]. The interest is largely due to anecdotal claims that this footwear modality is more *natural* and can therefore reduce injuries that may be caused by conventional running shoes (CRS) [5, 6]. With this increase in interest in barefoot running, footwear manufactures now produce *minimal footwear* that are marketed as having reduced conventional properties of the shoe such as stiffness, stack height, and shoe drop. Many runners are now attempting to switch to a more minimal running condition [4], and therefore have to undergo a transition to this footwear type. Injury risk may increase as a result of the transition because MFW lacks the conventional footwear properties of which the runner is now accustomed [7–9]. Runners attempting to transition to MFW must either adapt their running kinematics to suit a novel footwear condition or adapt the musculoskeletal system in order to accommodate different forces acting on the body due to changes in leg geometry/loading and footwear protection. The success and methods of how habitually shod runners can transition to MFW remain to be determined, but there is a growing body of literature that has investigated this problem (e.g. [8, 10, 11]).

Despite numerous authors and clinicians attempting to develop a *safe* transition schedule to MFW (e.g. [8, 10, 11]), there currently lacks any consensus of the methods that should be employed to make this potentially risky change in footwear modality. The methods employed vary in the amount of exposure to MFW, the duration of the transition period, and the control of other running volumes during this time. In addition, despite numerous authors making the suggestion that injury preventative exercise should be included in this transition [12, 13], these modalities are rarely included. An additional argument is that “the way that one runs is more important than what is on ones feet” [14], and therefore, clinical practice should consider the inclusion of gait-retraining elements to promote the likelihood of *desired* changes in running form. These gait-retraining changes, such as adopting a non-rearfoot strike and increasing stride frequency, have been found to have positive effects on a risk of injury. For example, adopting a forefoot strike reduces pain and disability in runners with chronic exertional compartment syndrome [15] and increasing step frequency has favourable effects on ground reaction force variables associated with tibial stress fracture [16]. Finally, the overall question that remains is whether there is any benefit of making this transition which may be potentially injurious for habitually shod western runners, who do not display the robustness and physically active background as our hunters gather ancestors of whom these theories of proposed benefits have been developed [17]. In other words, do we have the evidence that making a transition to MFW is worthwhile in a habitually shod and somewhat physically inactive [18] population?

Therefore, the purpose of this review is to systematically examine and report the methods that have been employed in the literature to transition to MFW, and the outcome of these studies with regard to injury and performance in runners. In addition, MFW transition guidelines for future clinical practice will be presented based on observations from this review and current practice.

## Methods

### Review Method

A systematic database search was employed using PubMed online as the primary database. In addition, Google Scholar and Scopus were also examined as complimentary databases. The terms “(Transition OR Habituation OR Training) AND (Minimalist OR Simulated Barefoot OR Barefoot Running Shoes) AND (Shoes OR Footwear) AND (Running)” were employed in the search strategy. The search was performed in February 2017. The literature search and screening of the abstracts were completed by the authors independently. Papers that both authors judged to meet the following a priori conditions and PICOS inclusion criteria were included and read in full: (1) the methods included individuals with previous running experience, of which their experience level was clearly reported; (2) the study prescribed specific details for transitioning to MFW including the proposed exposure to MFW, either within the paper or as an available resource; (3) the study included the use of “true minimal” shoes, based on the definition “Footwear providing minimal interference with the natural movement of the foot due to its high flexibility, low heel to toe drop, weight and stack height, and the absence of motion control and stability devices” [19]; and (4) only longitudinal prospective studies were included; (5) only full-length articles were included. The review procedure was based on guidelines from the PRISMA statement [20]. Both authors assessed the risk of bias for all included articles with a modified version of the Downs and Black Quality Index [21], used previously for systematic reviews in this area [22]. Definitions of *levels of evidence* were guided by Hall et al. [23]. The number of high-quality studies that examined the same variable and found a similar outcome decided the following levels: strong, *n* ≥ 3; moderate, *n* = 2; or limited, *n* = 1. Limited (*n* = 2 + studies) and very limited (*n* = 1 study) evidence was reported for low-quality studies with similar outcomes. Quality was assessed by the risk of bias score from the Downs and Black Quality Index, where studies that scored from 0 to 6 were classified as “high risk of bias” and very low quality, from 7 to 13 as “moderate risk of bias” and low quality, and from 14 to 20 as “low risk of bias” and high quality.

### Data Extraction

Relevant articles were fully examined for the following pre-determined areas of interest: participant information, the use of control or CRS groups during the transition duration, the period of the transition, the MFW implemented, the use of a log to record/monitor training during this transition, the schedule of the transition and how exposure to MFW was managed (calculated using the highest possible exposure within the group), the use of any preparatory exercises for the potential reduction in injury risk, the use of gait retraining in the transition, the injuries experienced by participants in both the intervention and control or CRS groups, and the rate of attrition (i.e. the amount of drop outs, considering injuries and other reasons). In addition, the main outcomes of the included studies were also reported, in order to summarise the evidence for potential positive/negative outcomes from a MFW transition. Given the purpose of examining the effects of a MFW transition, the results were focused on the changes that occurred because of the MFW transition rather than a comparison between MFW and CRS. Changes in outcome variables observed during the transition period in control or CRS groups were also given, but specific analysis as to the degree of change in CRS relative to MFW was not performed for all outcomes. Due to the potential for injury following a MFW transition [7–9], a direct comparison between MFW and CRS was performed for injury incidence only.

### Statistical Considerations

The prescribed exposure per running session or per week was mixed between percentages of typical running volume, by absolute miles per bout or per week, or by absolute time running in MFW. Therefore, in order to compare exposure across studies, we assumed an average running pace of 5 min/km to calculate the percent of the reported regular training that was performed in the MFW during the different phases of the transition period. Pooling was performed where studies investigated the same outcome measure with comparable methodology. Subject data was only included once in the pooling for studies by the same lab group that were conducted on the same subjects (e.g. [11, 24, 25]). Averages of values across papers did not include papers where the metric was not reported (e.g. attrition). A meta-analysis was performed on the injury occurrence only as there was insufficient data of similar outcome measures for meta-analyses of other variables to be undertaken. Most papers did not include a definition for *injury*. Therefore, for the purpose of the meta-analysis, an injury had to be symptomatic and resulted in missed training.

## Results

**Fig. 1:**
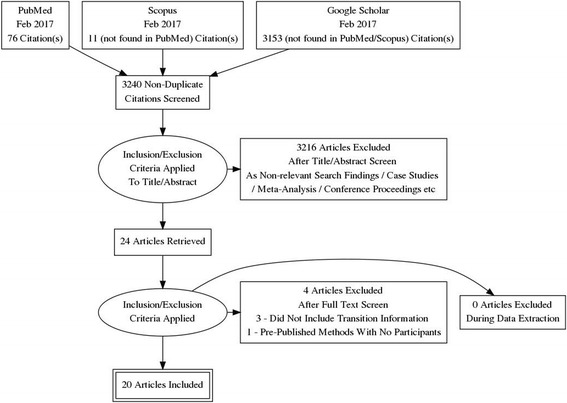
PRISMA study selection flow chart for the review. The relevant, non-duplicate citations were screened and included in the review if (1) the methods included individuals with previous running experience, of which their experience level was clearly reported; (2) the study prescribed specific details for transitioning to minimal footwear including the proposed exposure to minimal shoes; (3) the study included the use of “true minimal” shoes, based on the published definition; and (4) the study was longitudinal and prospective

The literature search returned 76 relevant articles and an additional 11 articles found in Scopus but not PubMed. The search in Google Scholar returned 3153 results not found in either Scopus or PubMed. The reference lists of initial articles were also screened for any relevant articles not found in the PubMed or Scopus searches. Three additional articles were sourced and considered for inclusion in the review. Twenty papers were included in the final review after screening each article for inclusion criteria (a study selection flow chart is presented in Fig. 1). A summary of the predetermined areas of interest is provided in Table 1, and the results of the Downs and Black Quality Index can be observed in Table 2. Seven studies included a control group who ran in their own running shoe for the training portion of the study [11, 24–29], five studies included a CRS group for which the shoes were provided [8, 30–33], and eight studies included only a MFW group [7, 9, 31, 34–38]. Some studies included groups that did not meet the guidelines for inclusion, such as a partial minimalist group [31], a barefoot group [36, 39], and walking groups [26]. Only the groups from these studies that met the guidelines for review were included in the analysis.

**Table 1:** A summary of key factors from the research papers included in this review. Studies are ordered from the shortest to longest transition period. Age ranges estimated from mean ± 1 SD are given because not all studies reported the mean age of participants

Source	Participant’s information	Groups (size of final *N*)	Transition period	Transition footwear	Training log used	Transition schedule (in MFW) [week]	Exercises included	Gait retraining included	Main study outcomes	Injuries experienced	Participant attrition
Willson et al. [9]	*N* = 19 Female, 18–35 years Running > 24 km/week	MFW (*N* = 17, tested in MFW and CRS)	2 weeks	Vibram FiveFingers (Bikila)	No	20 min, 3 times a week [1, 2] No other training	No	No. of participants informed that they were “not compelled to continue with a rearfoot strike pattern”	Runners that retained a rearfoot strike (9 of 12) showed 3 times greater LR in MFW vs. those who converted to non-rearfoot strike No change in kinetics over time across all participants	1 injury—lateral knee pain (was 1 of 2 that dropped out of study)	2/19 = 11%
Warne and Warrington [38]	*N* = 15 Male Well-trained runners, 19–29 years > 50 km/week	MFW (*N* = 15, tested in MFW and CRS)	4 weeks	Vibram FiveFingers (classic)	No	2 × 15 min [1], increase to 3–4 × 30 min [4]	Calf raises, golf ball rolling on the foot sole	No	1.05% more economical in MFW at pre-tests (ns), 6.9% at post-tests	Not reported	None = 0%
						Maintained total volume (substituted some CRS volume for MFW)					
Warne et al. [7]	*N* = 10 Female, 19–23 years Running average 45 km/week	MFW (*N* = 10) tested in MFW and CRS)	4 weeks	Vibram FiveFingers (KSO) (lab testing performed in Vivo Barefoot EVO)	No	3 × 5–8-min barefoot activity [1], 3 × 10–15 min [2], 3 × 20–25 min [3], 3 × 30–35 min [4] Maintained CRS volume Used grass and pavement	Foot sole and calf rolling, ankle mobility, calf raises, toe grabs, static balance	Shorten stride and increase cadence, run light and quiet, non-rearfoot landing, upright posture Encouraged for MFW and CRS	Reduction in plantar forces at post-tests in both MFW and CRS Higher mean and regional pressures in MFW vs. CRS	None	None = 0%
Bellar and Judge [34]	*N* = 13, 7 male, 6 female, 21–23 years Distance not reported Could run for 30 min continuously	MFW (*N* = 13, tested in MFW, barefoot and CRS)	5 weeks	Kigo Edge/Drive	Yes	5 × 30 min running/week, 1 of these in MFW and others in CRS [1], progress to all 5 in MFW [5]	No	No	3% improved running economy pre to post, likely a training effect	Not reported	None = 0%
Warne et al. [8]	*N* = 28 Male, 25–43 years Running > 40 km/week	MFW (*N* = 12, tested in MFW and CRS) CRS (*N* = 12, tested in CRS)	6 weeks	Vibram FiveFingers (KSO)	Yes	3 × 5–8-min barefoot activity [1], 3 × 10–18 min [2], 3 × 25–28 min [3], 3 × 30–35 min [4], 2–3 × 40–45 min [5, 6] Maintained CRS volume Used grass and pavement	Foot sole and calf rolling, ankle mobility, calf raises, toe grabs, static balance	Shorten stride and increase cadence, run light and quiet, non-rearfoot landing, upright posture	33% reduction in loading rate in the MFW group after transition Loading rate significantly higher in MFW vs. CRS at pre-tests	2 injuries in the MFW group (hamstring tear, calf tear) No injuries in the CRS group	4/28 = 14% (2 in the MFW group due to injury; 2 in the CRS group lost to follow-up)
Khowailed et al. [35]	*N* = 12 Female, 23–29 years Running average 25 km/week	MFW (*N* = 12, tested in MFW and CRS)	6 weeks	Vibram FiveFingers (Bikila)	No	3 × 5–8-min barefoot activity [1], 3 × 10–15 min [2], 3 × 20–25 min [3], 3 × 30–35 min [4] Maintained CRS volume Used grass and pavement	Running form drills, proprioceptive exercises, flexibility, strength, polymeric activities	Shorten stride and increase cadence, run light and quiet, non-rearfoot landing, upright posture Encouraged for MFW and CRS	Reduced loading rates and impact peak in transitioned MFW vs. CRS Decreased tibialis anterior activation and increase gastrocnemius activation with habituation to MFW	Not reported	Not reported
Moore et al. [36]	*N* = 10, 9 male, 1 female, 20–22 years 5–16 km/week	MFW (*N* = 10, tested in MFW and CRS)	7 weeks	Vibram FiveFingers (Komodo Sport)	No	Exercises only [1, 2], 20% progression in MFW per week [3–7] Not specified if participants maintained CRS volume	[1–2 only] heel raise, toe grip, dorsiflexion and plantar flexion, toe spread, exaggerated eversion and inversion, towel grabs	No	Higher peak pressures, loading rate and impact peak in MFW and barefoot vs. CRS However, loading rate and peak pressures decrease as a result of the transition in all footwear types	None	None = 0%
Warne et al. [32]	*N* = 23 Male, 33–53 years Running average 52 km/week	MFW (*N* = 12, tested in MFW and CRS) CRS (*N* = 8)	8 weeks	Vibram FiveFingers (KSO)	Yes	3 × 5–8-min barefoot activity [1], 3 × 13–18 min [2], 3 × 25–28 min [3], 3 × 30 min [4], 3 × 35 min [5, 6], 3 × 45 min [7, 8] Maintained CRS volume Used grass and pavement	Foot sole and calf rolling, ankle mobility, calf raises, toe grabs, static balance	Shorten stride and increase cadence, run light and quiet, non-rearfoot landing, upright posture	No change in running economy during transition	1 injury in the MFW group (metatarsal stress fracture) No injuries in the CRS group	3 of 23 = 13% (1 in the MFW group due to injury, 2 in the CRS group lost to follow-up)
Johnson et al. [11]	*N* = 44, 18–32 years Running average 24–48 km/week	MFW (*N* = 18)	10 weeks	Vibram FiveFingers (not specified)	Yes	1.6–3.2 km [1] + 1.6–3.2 km/week [2, 3] Then increase as tolerated Maintained CRS volume	No	No	Abductor hallucis cross-sectional area significantly increased in the MFW group, but no difference in size for the 3 other muscles tested	The same participants as Ridge et al. [24]	7 of 44 = 16% (non-compliance)
		Control (*N* = 19)									
Ridge et al. [24]	*N* = 43, 21 male, 15 female, 19–32 years Running 24–48 km/week	MFW (*N* = 19) Control (*N* = 17)	10 weeks	Vibram FiveFingers (not specified)	Yes	1.6–3.2 km [1], 1.6–3.2 km × 2 [2], at least 4.8 km [3] Then increase as tolerated Maintained CRS volume	No	No	Increased risk of stress fracture and bone marrow oedema in the MFW group following transition	10/19 classified as injured in the MFW group based on imaging results, and 2/19 of these with diagnosed stress fractures	7/43 = 16%
										No injuries or oedema in the control group	
Ridge et al. [25]	*N* = 25, 14 male, 11 female, 22–34 years Running 24–48 km/week	MFW (*N* = 10, tested in MFW and CRS) Control (*N* = 15, tested in MFW and CRS)	10 weeks	Vibram FiveFingers (not specified)	Yes	1.6–3.2 km [1], 1.6–3.2 km × 2 [2], at least 4.8 km [3] Then increase as tolerated Maintained CRS volume	No	No	Both groups improved RE over time, no interaction reported	The same participants as Ridge et al. [24]	6/25 = 24% (due to injury)
Ryan et al. [31]	*N* = 103, 39 male, 64 female, 19–50 years Able to run 60 min	MFW (*N* = 35) CRS (*N* = 32)	12 weeks	Vibram FiveFingers (Bikila)	Yes	1 week “break-in period” [1], 10% of volume in MFW [2], up to 58% [10] Then increase as tolerated Gradually increased running volume from 160 to 225 min until a 2-week taper [11, 91], leading into a 10-km event Included a longer run and interval training each week All training controlled	No	No	23% injury rate over 12 weeks in all participants No significant difference in injury comparing MFW and CRS Increased calf/shin pain in MFW	7 injuries in the MFW group (specific injuries not reported) 4 injuries in the CRS group (specific injuries not reported)	12/103 = 12% (lost to follow-up)
McCarthy et al. [28]	*N* = 30 Female,18–35 years Running > 15 km/week	MFW (*N* = 9, tested barefoot and CRS) Control (*N* = 10, tested barefoot and CRS)	12 weeks	Vibram FiveFingers (Classic)	Yes	Walking [1], 5-min walk, 1-min jog × 3, × 3/week [2], 3 × 5 min/week + 5 min/week [3–8], 1-day rec between [9–11, 91], individualised Maintained CRS volume	[1–2 only] from manufacturer’s recommendations Stretching calf muscles and self-massage of the calf and foot were also encouraged	Advised to avoid over-striding or use a rearfoot strike pattern No feedback provided	Shorter ground contact time, more anterior foot strike, greater ankle ROM, greater knee flexion at contact in the MFW group post-transition	4 injuries in the MFW group (calcaneal stress fracture [not related to running], hip and calf pain, 2nd metatarsal pain, metatarsal stress fracture)	11/30 = 37% (7/11 due to injury related to study)
										4 injuries in control (sciatica, anterior knee pain, ITB syndrome, back pain)	
Miller et al. [29]	*N* = 33, 17 male, 16 female, 24–36 years Running 48 km/week	MFW (*N* = 16, tested in CRS pre; CRS and MFW post) Control (*N* = 13)	12 weeks	New Balance (Road Minumus 10) or Merrel (Pace/Trail Glove) randomly paired	No	Comprehensive 12-week programme (controlling CRS volume also) Week 1: 2 1-mile runs in MFW, increase by 1 mile/week Increased to 3 MFW runs/week by week 4	No	Encouraged to maintain vertical trunk posture, use high cadence, and avoid over striding No foot strike instruction	Increases in foot musculature volume post-tests in both groups Greater stiffening of arch post-tests in the MFW group after outlier removed	No injuries in the MFW group	4/33 = 12% (3 due to injury, 1 lost to follow-up)
										3 injuries in control (Achilles tendonitis, plantar fascia tear, lower back pain)	
Joseph et al. [37]	*N* = 29, 7 male,15 female, 18–28 years Running 16–48 km/week	MFW (*N* = 22)	12 weeks of transition followed by additional 12 weeks of study participation	Newton Gravity	Yes	10% of total mileage in MFW for weeks 1 and 2 Increase by 10%/week until 100% reached in week 12 Maintained 100% through week 24	No	Instruction given for forefoot strike pattern, decreased stride length, increased stride frequency, forward trunk lean Video of running style provided	No change in plantar flexion force, Achilles tendon cross-sectional area, mechanical characteristics or material properties between baseline, 6, 12, and 24 weeks	4 injuries (exacerbated previous knee pain)	7/29 = 24% (7% relocation, 14% knee pain, 3% non-compliance)
						Other volume maintained in CRS until 100% in MFW					
Dubois et al. [30]	*N* = 26, 8 males, 18 female, 18–55 years Running distance not reported Could run 20 min continuously Running experience of one half or full marathon	MFW (*N* = 11) CRS (*N* = 9)	16 weeks (pilot study)	Inov-8 (F-lite 195/Bare X-lite 150/Road X-lite 155), Mizuno (Wave Universe), Saucony (A5)	Yes	Comprehensive 16-week programme Progressed from 3 to 7 × 1 min [1], to one half marathon [15] All training in MFW	No	No	15.4% drop out rate after randomisation, *N* too small to detect the injury difference between groups	3 injuries in the MFW group (metatarsal, stress fracture, iliotibial band syndrome, plantar fasciitis) 3 injuries in CRS (low back pain, medial tibial stress syndrome × 2) Missed training due to pain the same in groups	6/26 = 23% (2 prior to randomisation, 4 during study)
Campitelli et al. [26]	*N* = 48 (96 ft), 25 male, 16 female completed study, 20–33 years, no barefoot or MFW experience Control group had to be running 10–40 miles/week	MFW (*N* = 12) Control (*N* = 12)	24 weeks (assessments at 0, 12, and 26 weeks)	Vibram FiveFingers (Bikila)	No	Training restricted in the MFW group only: 4 training sessions per week; increased mileage or time in MFW by 10% each week starting with 0.25 time/mileage restriction week 1 up to 6.0 in week 24 CRS worn for any additional time/mileage	No	Brochure on proper running form (not specified)	Increase in abductor hallucis longus thickness between 0 and 24 weeks in the MFW group No difference in thickness over the study period in the control group No group differences in muscle thickness	No injuries reported	7/48 = 15%
											(2/12 = 17% in the MFW group, 3/12 = 25% in the control group)
Azevedo et al. [39]	*N* = 34, 25 male, 9 female, 23–37 years Running 44–88 km/week	Barefoot (*N* = 6), MFW (*N* = 8)	6 months	New Balance (Minimus MR10BG)	Yes	3 training sessions per week in the MFW In each 2 months, a more “minimal” shoe was used Maintained CRS volume	No	No	In the MFW group, 6/14 participants dropped out due to pain/injury	6 injuries in the MFW group (“injury/pain”—specific injuries not reported)	20/34 = 59% (70% in the barefoot group, 30% in the MFW group)
										2 injuries in the barefoot group (“injury/pain”)	40% injury/pain, 40% time/place, 15% fear of injury, 5% accident
Chen et al. [27]	*N* = 47, 21 male, 17 female, 20–45 years Running average 26 km/week (MRS), average 35 km/week (CRS)	MFW (*N* = 20) Control (*N* = 18)	6 months	Vibram FiveFingers (not specified) Minimalist Index 92% [12]	Yes	Transition adopted from the Spaulding Natural Running Centre [13]	[1–3 only] 30× calf raises, dynamic balance, foot placement, calf/Achilles stretches	Land gently, with your foot relatively horizontal and under your hips (this will shorten your stride)	Increase in muscle volume in intrinsic and extrinsic foot muscles in the MFW group Muscle volume associated with compliance to MFW transition	No injuries	9/47 = 19% (8 conflicts, 1 lost to follow-up)
						Stage 0: pre-entry barefoot activity					
						Stage 1: walk and jog					
						Stage 2: jogging every other day					
						Stage 3: jogging multiple days					
						Stage 4: five loading days in 1 week					
						Stage 5: full activity					
						It was not clear what volume of CRS running was maintained					
Fuller et al. [33]	*N* = 61 All male, aged 18–40 Running at least 15 km/week Maximum 5k time = 23 min	MFW (*N* = 31)	6 months	Asics (Piranha SP4)	Yes	6 weeks of training standardised for both groups (long slow distance and intervals included) 5% of each run in MFW [1] maintained CRS volume Increase MFW volume by 5% each week until 100% MFW [19]	No	No	Shoe × body mass interaction for time to running-related injury	Training in MFW increased knee and calf pain; 11/30 (37%) in CRS became injured; 16/30 (51%) in MFW became injured Time to injury was not affected by shoe type In MFW, injury was statistically more likely with body mass > 71.4 kg	5/30 (17%) in the CRS group, 4/31 (13%) in the MFW group
		CRS (*N* = 31)									

*MFW* minimal footwear, *CRS* conventional running shoes

**Table 2:** Modified Downs and Black’s checklist results. The scale was composed of 20 items related to information reporting (items 1 to 9), external validity (items 10 and 11), internal validity (items 12 to 15), and selection bias (items 16 to 20). Each item was scored 0 to represent a high risk of bias or 1 to represent a low risk of bias. Studies that scored a total of 0 to 6 were classified as “high risk of bias”, from 7 to 13 as “moderate risk of bias”, and from 14 to 20 as “low risk of bias”

Checklist	Studies																			
	Wilson et al. [9]	Warne and Warrington [38]	Warne et al. [7]	Bellar and Judge [34]	Warne et al. [8]	Khowailed et al. [35]	Moore et al. [36]	Warne et al. [32]	Johnson et al. [11]	Ridge et al. [24]	Ridge et al. [25]	Ryan et al. [31]	McCarthy et al. [28]	Miller et al. [29]	Joseph et al. [37]	Dubois et al. [30]	Campitelli et al. [26]	Azevedo et al. [39]	Chen et al. [27]	Fuller et al. [33]
Is the hypothesis/aim/objective of the study clearly described?	1	1	1	1	1	1	1	1	1	1	1	1	1	1	1	1	1	1	1	1
Are the main outcomes to be measured clearly described in the Introduction or Methods section?	1	1	1	1	1	1	1	1	1	1	1	1	1	1	1	1	1	1	1	1
Are the characteristics of the participants included in the study clearly described?	1	1	1	1	1	1	1	1	1	1	1	0	1	1	1	1	0	1	1	1
Are the interventions of interest clearly described?	1	1	1	1	1	1	1	1	1	1	1	1	1	1	1	1	1	0	0	1
Are the distributions of principal confounders in each group of subjects to be compared clearly described?	0	0	0	0	0	0	0	0	0	0	0	0	0	0	0	1	0	0	1	0
Are the main findings of the study clearly described?	1	1	1	1	1	1	1	1	1	1	1	0	1	1	1	1	1	1	1	1
Does the study provide estimates of the random variability in the data for the main outcomes?	1	1	1	1	1	1	1	1	1	1	1	0	1	1	1	1	1	0	1	1
Have all important adverse events that may be a consequence of the intervention been reported?	1	0	1	0	1	0	0	1	1	1	1	1	1	1	1	1	0	1	1	1
Have actual probability values been reported?	1	1	1	1	1	1	1	1	0	1	1	0	1	1	1	1	1	0	1	1
Were the subjects asked to participate in the study representative of the entire population from which they were recruited?	0	0	0	0	0	0	0	0	0	0	0	0	0	0	0	0	0	0	0	0
Were those subjects who were prepared to participate representative of the entire population from which they were recruited?	0	0	0	0	0	0	0	0	0	0	0	0	0	0	0	0	0	0	0	0
Was an attempt made to blind those measuring the main outcomes of the intervention?	0	0	0	0	0	0	0	0	0	0	0	0	0	1	0	1	0	0	1	0
If any of the results of the study were based on “Data dredging”, was this made clear?	1	1	1	1	1	1	1	1	1	1	1	1	1	1	1	1	1	1	1	1
Were the statistical tests used to assess the main outcomes appropriate	1	1	1	1	1	1	1	1	1	1	1	1	1	1	1	1	1	0	1	1
Were the main outcome measures used accurate (valid and reliable)?	1	1	1	1	1	1	1	1	1	1	1	1	1	1	1	1	1	1	1	1
Were the participants in different intervention groups (trials and cohort studies) or were the cases and controls (case-control studies) recruited from the same population?	0	0	0	0	1	0	0	1	1	1	1	1	1	1	0	1	1	1	1	1
Were study subjects in different intervention groups (trials and cohort studies) or were the cases and controls (case-control studies) recruited over the same period of time?	0	0	0	0	0	0	0	0	0	0	0	0	0	0	0	1	0	0	0	0
Were study subjects randomised to intervention groups?	0	0	0	0	1	0	0	1	1	1	1	1	1	1	0	1	0	0	1	1
Was there adequate adjustment for confounding in the analyses from which the main findings were drawn?	0	0	0	0	0	0	0	0	1	0	0	0	0	0	0	0	0	0	0	0
Did the study have sufficient power to detect a clinically important effect where the probability value for a difference being due to chance is less than 5%?	1	0	0	0	0	1	0	0	0	0	0	1	1	0	0	0	1	0	0	1
Total	12	10	11	10	13	11	10	13	13	13	13	10	*14*	*14*	11	*16*	11	8	*14*	*14*

Low-risk studies are highlighted in italics

The participants ranged from well-trained to recreational runners, running anywhere from 15 to 88 km/week, and included both male and female participants (male, *N* = 342; female, *N* = 281). All studies included participants with no previous barefoot or minimalist experience at the start of the study. Inclusion criteria regarding previous running experience varied from as low as 4.8 km/week [36] or “being able to run 20 minutes” [34] to as much as 88 km/week [39]. The average reported running distance/week was 41 km. Only two studies had an experimental group with *N* > 30 [31, 33]. The transition period ranged from 2 weeks to 6 months, with the most common footwear brand used in the transition being the Vibram FiveFingers. Thirteen out of twenty studies included a training log to measure compliance to the transition schedule.

The transition schedule implemented in many of the studies involved a gradual increase in the exposure to minimal shoes over the transition period. The amount of training in minimalist shoes undertaken during the first week of the programme ranged from estimated mean of 8% (range 0–24%) of the participants’ regular training and increased to an estimated mean of 60% (range 30–100%) at the end of the transition schedule. However, Willson et al. maintained the same amount of MFW exposure each week for the short 2-week transition [9], three studies allowed training only in the MFW that increased gradually each week [31, 40], and three studies based on the same cohort [11, 24, 25] controlled exposure for the first 3 weeks and then allowed the participants to increase exposure as they saw fit. Participants maintained their normal total training volume in 14/20 studies; participants simply substituted some of their running in CRS with MFW incrementally over this period. However, several studies [29, 31, 33, 41] controlled the entire training schedule in both CRS and MFW or did not allow any other training than that completed in MFW [9, 30]. Several studies [7, 8, 27, 28, 31, 32, 35] also encouraged non-running activity in the first week of the schedule as acclimatisation for the novel footwear. Only 3/20 studies [7, 8, 32] prescribed a specific running surface, where it was recommended that both grass and road running was included.

Only 8/20 of the included studies implemented supplementary exercises performed two to three times a week. One out of these eight studies included a CRS or a control group, but exercises were only implemented to the MFW group [28]. Moore et al. [36] included these exercises for 2 weeks before the transition to MFW began. Two studies [27, 28] included these exercises during weeks 1–3 and 1–2, respectively. Supplementary exercises were similar across studies and included concentric/eccentric triceps surae strength work, self-myofascial release techniques of the foot and lower leg, intrinsic foot musculature exercises, ankle proprioceptive/balance work, and light plyometrics.

Nine out of twenty of the included studies used some form of gait retraining or advice as part of the MFW transition. The training or advice included some or all of the following: a more upright posture with hips forward, increased cadence (+ 10%), running “lightly and quietly”, and adopting a non-rearfoot strike pattern. One study [29] included the above gait retraining but provided no instruction on foot strike. Two studies [26, 28] provided recommendations but no direct feedback, and one study [9] informed the participants that changes happen but told them not to be *compelled* to make any changes. Most studies implemented gait retraining or advice through a deliberate instruction at pre-tests. No study reinforced, quantitatively measured or monitored, or provided ongoing feedback for the transition.

### Main Study Outcomes

Four studies investigated running economy, one of which included gait retraining [25, 32, 38, 41]. The one study that examined the combination of a MFW transition and gait retraining noted no improvement in running economy [32]. The three studies that did not include gait retraining demonstrated improvements in running economy in MFW of 8% after 4 weeks [38], 3.4% after 5 weeks [34], and 10.4% after 10 weeks [25]. The 3.4 and 10.4% improvements in running economy observed after the MFW transition [25, 34] were likely a training effect, given that the control or CRS group also improved running economy from pre- to post-transition (4.1% [25], 2.8% [34]). Therefore, there is currently limited evidence that a transition to MFW will improve running economy.

Running kinetics that are debated commonly in relation to injury development were examined in four studies. Only one of these studies included a separate control or CRS group [8]; the remaining studies tested all participants in all footwear conditions. Willson et al. observed no statistical change in loading rate (< 7.0%) during MFW running following a short 2-week transition [9]. In contrast, two studies observed significant reductions in a loading rate of 36.7 and 33.0% in the MFW condition from pre- to post-transition [8, 35]. However, it is not clear if the reduction in loading rate observed in Khowailed et al. [35] was a footwear effect or a result of changes to stride characteristics after the transition because runners were not assessed in the CRS condition post-transition. In addition, Moore et al. [36] reported decreases in average and instantaneous loading rate of 9.3 and 48.1%, respectively, and a 25.7–72.5% reduction in peak pressure in the MFW condition after a 7-week transition period. Peak pressure tended to increase in the forefoot region in two studies with a 4-week transition [7, 36] that was potentially related to the adoption on a non-rearfoot strike.

Muscle or tendon properties were assessed by 5/20 studies [11, 26, 27, 29, 37]. Two studies reported abductor hallucis cross-sectional area significantly increased within the MFW group by 10.4% after 10 weeks post-transition [11] and 18.8% after 24 weeks [26] but no significant change in this muscle was observed after 12 weeks in another study [29]. Increases in foot musculature volume and arch conformation post-tests in other MFW-transitioned groups were also supported by Chen et al. [27]. In Chen et al. [27], increases in leg and foot muscle volume was associated with compliance to the MFW transition; the greater the compliance, the greater the muscle volume increase. Comparisons between footwear conditions in some studies need to be interpreted with caution, given the differences in muscle thickness between groups at baseline and the potential differences in running volume between groups [26]. Joseph et al. [37] measured Achilles tendon cross-sectional area, material properties, and mechanical characteristics over the course of a 24-week transition to MFW and found no differences between time points for any of these variables. There is, therefore, limited evidence for increases in foot muscle size, but no evidence for Achilles tendon adaptations as a result of a transition to MFW.

### Injury and Attrition

Three studies did not report any injury information [35, 36, 41]. Twelve out of twenty studies compared the number of injuries sustained during the transition period between MFW and CRS groups; however, three studies [11, 24, 25] reported the same participants and, therefore, only the earliest publication is included here. Out of these ten remaining studies, five observed more injuries in the MFW vs. the CRS group [8, 24, 31–33], one observed more injuries in the CRS group vs. the MFW group [29], and four did not observe any difference in injuries between groups [26–28, 30]. Across these 12 studies, 35 related injuries were experienced in the 195 total participants transitioning to MFW (17.9 injuries per 100 participants). Twenty-five related injuries were experienced in the 187 total control or CRS participants (13.4 injuries per 100 participants). A chi-square test of independence indicated no significant difference in injury risk between groups (*p* = 0.219; effect size phi (*φ*)= 0.06 (very small); power = 0.23; odds ratio for injury in the MFW group = 1.174 [95% CI = 0.923 to 1.493]; odds ratio for injury in the CRS group = 0.828 [95% CI = 0.602 to 1.139]). However, an overall comparison between the MFW and CRS groups is limited, given 5/12 studies that reported injuries did not include a control or CRS group [7, 9, 36, 37, 39]. In addition, several studies did not report the specific injury and, therefore, analysis of injury type was not possible. Therefore, conflicting evidence exists for differences in injury when running in CRS vs. transitioning to MFW.

Two studies [11, 24] assessing bone marrow oedema reported numerous injuries in the MFW group; however, these were reported as asymptomatic (no missed training) and so should be interpreted with caution. The asymptomatic injuries were not included in the injury analysis of this study. In the study for which injury rate was examined as the primary outcome variable, no significant difference in injury was observed during a 12-week 10k training programme between MFW (seven injuries) and CRS (four injuries) groups [31]. However, the exact statistics for this difference were not supplied.

Greater knee and calf pain was associated with MFW after a 6-month transition, especially when running volume was greater than 35 km/week. Compared with the CRS group, the MFW group had a greater risk of injury development if body mass was above 71.4 kg (hazard ratio = 2.00; 95% CI = 1.10–3.66) [33]. Greater incidence of calf pain in the MFW was also reported after a 12-week transition [31].

The rate of attrition ranged from 0 to 59% over the 19/20 studies that reported dropout data. The calculated average across all studies that reported dropout was 16 ± 13%, including those that reported no attrition (4/20).

## Discussion

The purpose of this review was to systematically examine the literature that has implemented a transition to MFW, in order to compare the methods of transitioning and the outcomes of a MFW transition. The main findings included a large variation in the methods employed to transition participants to MFW. Some potential benefits of this transition were observed in some but not all studies. In particular, we observed limited evidence for an increase in the muscle cross-sectional area of the foot, limited evidence for an improved running economy, and limited evidence for a reduction in loading rate. However, elevated loading rate, bone marrow oedema, and plantar forces in the early stages of the transition should lead to caution in the exposure to MFW, initially. The directions of the findings, however, were not consistent between studies. For example, some studies found an improvement in running economy in MFW after the transition [25, 34, 38] whereas another found no difference [32]; average loading rate decreased in MFW after the transition in some studies [8, 36] but not others [9] or an increase in instantaneous loading rate was found [36]; and mixed results were found regarding the injury rates experienced in MFW during the transition period. Methods and length of transition period, inclusion of exercises and/or gait retraining, and other methodologies may explain these differences in findings between studies. In addition, the quality index assessment identified only 5/20 studies being a high level and low risk of bias, and therefore, future studies should attempt to conduct more stringent research in this area.

### Methods of Transitioning to MFW

The studies included in this review used a wide range of strategies for transitioning to MFW, which makes it difficult to suggest a method that should be adopted in practice. Most studies began the transition with a period of walking and/or fewer than 10 min of running in MFW for at least the first week whereas only a few studies prescribed a higher initial exposure to MFW. There does not appear to be a clear relationship between transition method and study outcome or injury rate. There is likely an interaction between several factors, such as running experience, initial exposure to MFW, and length of transition period. Various transition durations or different exposure methods have not been compared within any studies to date, and this therefore represents a future potential research avenue.

It is unclear if the protocols of the reviewed studies resulted in a full or complete transition to MFW. Only a few studies required at least one running session to be completed entirely in MFW by the end of the transition period. A full transition (i.e. 100% of weekly running volume) to MFW was accomplished by three studies during the last week of the transition [33, 34, 37] or not specifically stated in other studies. It is possible that running in both MFW and CRS causes motor interference that prevents a true MFW-adapted gait pattern from emerging. Therefore, more research is needed to distinguish whether the altered gait resulting from MFW transition is a temporary performance of an observable behaviour or a permanent, learned motor skill.

To determine when someone is *fully transitioned* to MFW is entirely problematic, and therefore, a clear timeline cannot be established based on the current literature. Willson et al. suggested that a 2-week intervention is not likely to result in a natural conversion in foot strike pattern [9]. Giandolini et al. observed that at least a 1-month intervention is required to adopt a new kinematic pattern in response to MFW; however, these authors also observed a regression back to pre-intervention gait mechanics after 2 months [42]. The transition timeline is, certainly, an individual response that cannot and should not be universalised from a cross-sectional study. What is the operational variable that should be used to define if someone is fully transitioned? Should establishment of an appropriate timeline consider bone oedema reduction, running economy plateaus, plateaus in loading factors, or a subjective means of comfort/pain in MFW? These are all important factors that together determine the reasons for changing footwear initially, but given the individual responses to all factors, as well as the difficulty in combining all factors in the determination of being *transitioned* to MFW, we may have to accept that no clear timeline can be established using a single scientific method. With regard to recommendations for the duration of a transition to MFW that should be used in clinical and scientific practice, we suggest that a transition period of no less than 4–8 weeks should be used because of general muscular adaptation to training, taking this period of time [43].

The exposure to MFW was also extremely varied across included studies. There are three factors that should be considered in this regard: (1) the initial exposure, (2) the increase in exposure each week, and (3) the desired amount of total running volume in MFW by the end of the transition. Importantly, several of the included studies [7, 28, 32, 35, 38] implemented a period of preparation before this initial exposure, and this has also been recommended in the previous literature [44–46]. Given the dramatic change in the demand of the foot structure and musculature with MFW use, a period of preparation could include some light walking and every day, non-uniform loading whilst wearing MFW or going barefoot may be of benefit before any running activity is begun [44–46]. In addition, foot muscle size may be important for transitioning safely [11]. However, there are currently no studies that have evaluated whether this preparatory phase has any influence on overall injury incidence compared with a group that does not undergo a preparatory phase. As with many components of any novel transition requiring a new or different neuromuscular control pattern and altered loading, many practices are based on *common sense* moreso than evidence-based practice and should be interpreted with caution.

**Fig. 2:**
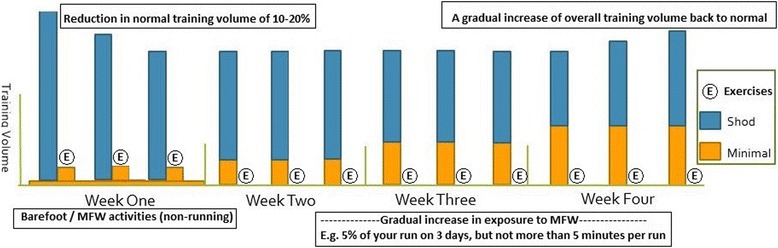
A simple example of how one might structure the initial stages of a MFW transition. Note that it is not intended that a MFW transition takes place over just 4 weeks

The initial running exposure to MFW varied in the literature from 0 to 24% of typical running volume in the first week. Whilst the “10-percent” rule of increasing training volume to prevent injury has recently been debunked [47], a safe amount to increase the specific exposure to MFW per week has yet to be determined because differences in transition programmes have not been compared between groups within the same study. The 5% guidelines presented by Fuller et al. [33] appear to be an appropriate start point in this regard for increasing MFW exposure. In addition, the total amount of running volume to be completed in participant’s regular running shoes whilst making this transition should be considered, as it is important that runners maintain a normal volume of running training to maintain cardiorespiratory fitness. This notion is reflected in the literature, where 14/20 included studies allowed participants to complete their typical total weekly training volume by increasing the percentage of this training per week in MFW and decreasing the percentage of training performed in CRS. However, given what has been observed with increases in bone marrow oedema when running initially in MFW, we suggest that the initial overall running volume is decreased in the region of 10–20% in the first 2 weeks (Fig. 2), in order to reduce the risk of this bony injury from unfamiliar repetitive loading. This suggestion is based on consistent evidence that training volume is related to running injury risk [48, 49].

**Fig. 3:**
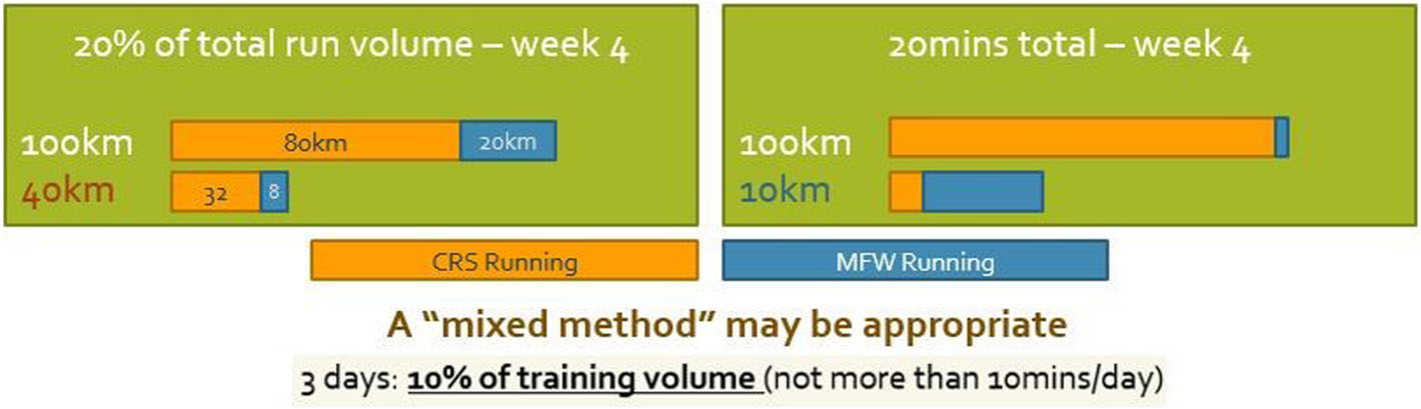
An example of the two common prescription methods for MFW exposure (distance vs. time). On the left, it is apparent that athletes running a high training volume (100 km/week) would require a full 20 km of running in MFW to meet the criteria which would present a possibly dangerous exposure. On the right, however, an athlete running just 10 km/week would find themselves running almost entirely in MFW if asked to run 20 min in this footwear, which might also be dangerous, given their low running exposure initially. Therefore, a *mixed method* is suggested

Almost all of the authors dictated a gradual increase in exposure to MFW each week throughout the programme; however, three studies based on the same cohort [11, 24, 25] only controlled exposure for the first 3 weeks before allowing participants to increase MFW volume to whatever amount they saw fit. Interestingly, these studies reported consistent injuries and high rates of bone marrow oedema when compared to the remaining literature, and suggested a careful progression to MFW should be prescribed at all times. Two studies, which were not included in the review due to a lack of specific prescribed transition protocol, also reported higher rates of injuries (i.e. 86–90%) [50, 51] than studies implementing a specific transitioning protocol that were included in this review. There were two methods of prescribing exposure to MFW in the reviewed studies: an absolute value (e.g. 10 min per day) or a relative value (e.g. 10% per day). As can be observed from Fig. 3, there can be issues with regard to over-exposure and increasing injury risk when incorporating only one prescription method. Therefore, we suggest a hybrid approach—e.g. “10% of your daily running volume, up to a maximum of 10minutes”—that is increased by 5–10% per week. One important point is that a minimum of 4 min during any run has been suggested to optimise the foot-surface interaction [52], and so initial increases should be no less than this duration. A suggested initial transition schedule for runners is presented in Fig. 2; however, further research is needed to determine if the initial transition schedule should be tailored for runners of different experience levels.

The increase of exposure during the course of the transition to MFW is critically important. The surveyed literature has used progressive exposure programmes. However, this exposure of MFW is not individually prescribed and, therefore, does not take into account individual risk factors for injury. A potential future method of prescribing MFW should take into account a number of known risk factors (such as those presented in Table 3) before determining the initial and overall exposure. For example, females have been found to be at greater risk of experiencing bone marrow oedema when transitioning to MFW [11] than males and males with a body mass greater than 85.7 kg have a greater risk of developing a transition injury [33]. Therefore, females and heavy males should perhaps consider a more conservative programme. Using a screening protocol to determine high-, moderate-, or low-risk participants, clinicians may be able to individually prescribe the exposure to MFW. This theory requires further investigation.

**Table 3:** A list of possible risk factors for injury in runners. Evidence is only from systematic reviews and meta-analysis and does not include weak/limited evidence

Source	Risk factors for injury
Zadpoor and Nikooyan [92]	Higher loading rate
van Mechelen [59]	Running inexperience
	Previous injury
	Running to compete
	Excessive distance/week
Tonoli et al. [93]	Younger
	Previous injury
	Less running experience
Van Gent et al. [49]	High mileage
	Previous injuries (BUT this was a protective factor for knee injuries)
Yeung and Yeung [94]	High mileage
	High frequency of training
	High distance
Chuter and Janse de Jonge [95]	Excessive foot eversion (but may be a protective factor for stress fractures)
	Poor “core” stabilisation
Murphy et al. [96]	Regular competition
	Running on artificial turf
	Previous injury
	Specific to stress fractures
	Pes cavus
	Excessive foot inversion
	Decreased bone mineral density
van der Worp et al. [97]	History of previous injury
	Having used orthotics/inserts
Hulme et al. [98]	History of previous injury
	Irregular and/or absent menstruation in females = stress fracture risk

The final question with regard to exposure to MFW is how much should participants be running in MFW at the end of the intended transition programme? Do we really need to be running 100% in MFW? Of the studies in this review, very few specifically reported or prescribed that participants ran 100% in MFW by the end of the transition. There is evidence that running in multiple different pairs of shoes can reduce injury risk [53], most likely due to variations in repetitive stress as a result of changes in shoe cushioning properties as well as biomechanical variations in the running gait [54–58]. The same concept applies to a variation of the running surface (of which only three studies have prescribed [7, 8, 32]); a constantly changing underfoot environment will reduce the risk of repetitive loading on the same structures, and variability has been suggested to potentially reduce the risk of injury [54–58]. In addition, there is no evidence that surface hardness is linked to increased injury in runners and, therefore, hard surfaces should not necessarily be avoided [59]. Therefore, if running in MFW is desired either by the patient or prescribed by a clinician, it is suggested that some proportion of running takes place in different footwear classifications, or even a proportion of entirely barefoot, and on numerous different surfaces. By developing the ability to deal with multiple stressors in a variety of environments, we may be able to develop more resilient runners and combat the dramatic injury rates seen today [60, 61].

### Injury Prevention Exercises

It has been suggested by some authors that a barefoot or MFW transition should include injury prevention exercises [12, 13]; however, only 8/20 studies in this review included this element. It is understandable that research scientists attempt to control for confounding factors and therefore do not include injury prevention exercises as they may be responsible for some changes to the dependent variable. However, the applied sciences should consider examining the combination of injury prevention exercises and a MFW transition to a greater extent, since their inclusion better reflects real-world practice and may also play a role in offsetting the potentially high injury risk of this transition. Indeed, foot muscle size has been found to be important for transitioning safely [11], and the use of a foot “doming” exercise was also found to increase foot muscle size [62]. In addition, a structured exercise programme can potentially reduce injury in runners [63–65], although the direct effect of the inclusion of injury prevention exercises in a MFW transition has not yet been examined with regard to injury risk. This programme should not only increase neuromuscular conditioning but should also prepare for and combat the increase in delayed-onset muscle soreness and tightness that is consistently observed in the initial stages of MFW use [9, 31, 66, 67]. Suggested exercises have been included in Table 4.

**Table 4:**
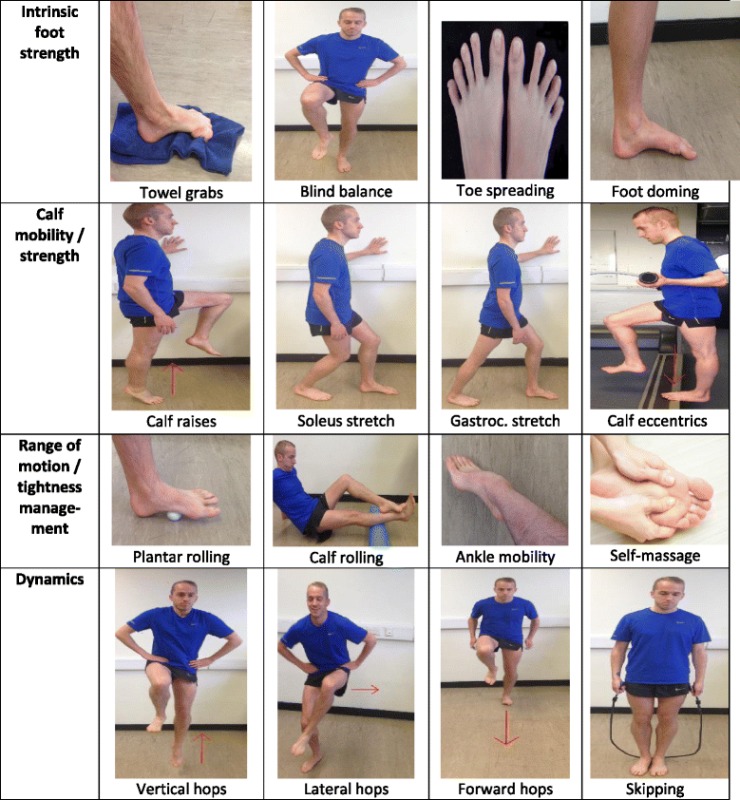
Simple injury prevention exercises suggested for a minimal footwear transition. Note that these exercises require systematic evidence for their role in reducing injury risk. Exercises should be included several times a week, and the *dynamic* exercises should only be included after a minimum of 2 weeks due to the increased load and plyometric nature of these exercises. Sets/reps should be decided upon by a trained professional in line with the FITT-VP principles (frequency, intensity, time, type, volume, progression)

### Gait Retraining

The inclusion of gait retraining was observed in 7/20 of the final studies in this review. The reason for including this component was debated in Warne et al. [8]. In short, the many runners choosing to use MFW do so in the hope of reduced injury or improved performance largely in the form of changes to running mechanics (to a more “barefoot” style). Since many runners have been observed to retain a typically “shod” running style even in MFW [7–9, 32, 38], these runners may benefit from the inclusion of some simple gait retraining. For example, a rearfoot strike in minimalist results in significantly higher loading variables [9, 68, 69].

Common gait-retraining instructions in the literature include adopting a non-rearfoot strike [6, 66, 70–72], increasing cadence [73–75], and using gait-retraining *packages* such as “Pose” [76–78] or “Chi” running [76]. Again, including gait retraining to adopt these characteristics may apply moreso to applied science where the combination of MFW use and simple gait retraining is commonly observed in the real world, as opposed to controlled research science where confounding factors may reduce the ability to differentiate one effect from the other.

The above gait changes are commonly desired by the participant because they have been related to a reduction in factors related to injury [15, 66, 70, 73, 74, 78, 79]; thus, their inclusion may be recommended for clinical practice. The effect of adopting a non-rearfoot strike pattern during a MFW transition has not been investigated specifically with respect to reducing injury incidence. If gait retraining is adopted by clinical practice, it is important that the prescription follows motor learning principles both for uptake and retention. This includes limiting the amount of cues, giving clear verbal and visual instruction, and providing interpretable visualisation queues [80]. Simple suggestions for gait retraining have been presented in Table 5. It should be noted that the authors acknowledge the benefit of the use of augmented or biofeedback practice for the enhanced retention of gait retraining (e.g. [81, 82]); however, this service and technology is not accessible to the vast majority or runners, hence simple universal guidelines being presented here. Finally, if the prescription of MFW is implemented for improvements to running economy, please note that gait retraining has been found to have no effect, or even worsen running economy both with and without the use of MFW in the literature [32, 78, 83–86].

**Table 5:** Simple gait retraining queues suggested for a minimal footwear transition

Gait retraining change	Visual/feedback queue	Evidence for effect
Adopt a non-rearfoot strike pattern^a^	“Imagine you are running on sharp, hot stones”	[6, 15, 66, 70, 72]
Increase stride frequency (10%)	Use of a metronome	[57, 73, 74]
Land more quietly	“Imagine running whilst sneaking up on someone”	[67]

^a^Note that adopting a non-rearfoot strike can increase ankle work [99–101]

### Study Outcomes

The body of evidence suggests limited evidence for positive benefits of transitioning into MFW for running economy. It appears that kinetic loading factors such as the loading rate are potentially reduced with exposure to MFW between pre- and post-tests but not compared with CRS [8, 35, 36]; therefore, higher initial loading rate and plantar pressures may increase injury in this footwear condition in the early stages [8, 36]. This hypothesis requires further investigation as there is currently no high-level evidence of increased injuries in this period. There also appears to be limited evidence of increases in muscle cross-sectional area and muscle volume in the literature examining a transition to MFW [11, 27, 29]. However, direct links between these changes in muscle volume and injury risk remain to be determined.

### Injury Rate

The injury risk when transitioning to MFW has been suggested to be increased during the initial period of change [7–9, 11, 24]. In particular, metatarsal stress fractures in MFW have been reported in the studies in this review, as well as in several case studies on the topic [87, 88]. However, longitudinal prospective studies examining injury incidence comparing habitual CRS and MFW runners, as well as those during the transition period, are lacking. Only one prospective study has compared injury incidence between habitually shod and habitually barefoot/MFW runners [89] and observed no difference in injury risk after controlling for mileage. Interestingly, this study claimed that most runners in the barefoot/MFW group had only been running in this footwear type for a reasonably short period of time (1.65 ± 1.32 years), and therefore, many may still be considered *transitioning* participants. Although this paper was an important contribution to the literature, it was a survey study and, thus, the potential of recall bias may have been a factor in the results. Two studies included in this review identified no differences in injury between the CRS and MFW groups following training for a 10k race over 12 weeks in recreational runners [31] or following a 6-month training period in more experienced runners [33]. However, a risk of injury when transitioning to MFW may be increased in heavier runners [33]. The difference in time running in MFW vs. CRS has not been accounted for in the present review, but the injury results support the previous research in which injuries between the MFW transitioning group and the control/CRS group was not significantly different although the statistical power of this analysis was low. Therefore, unless high-level evidence emerges, we have no reason to believe that the injury rates are any higher either during a transition to MFW or habitually wearing MFW when compared to running in CRS. There may be specific differences in injury trends amongst groups, such as increased foot injuries in the MFW group [89], but not in the rate of injury.

### Future Recommendations

Future recommendations for research on the transition to MFW could benefit from some observations of this review. Specifically, the use of a logbook to document the rate of adherence to both the MFW exposure as well as other components such as gait retraining and injury prevention exercises is warranted. Only 14/20 studies included this log, and therefore, information on participant compliance with the schedule is often absent. Secondly, attempts to isolate key confounding factors should be made by using control groups running in their own CRS, rather than a new CRS for which they may not be accustomed, alongside the transitioning groups. In this regard, the inclusion of extra elements such as gait retraining and exercises can be examined in isolation, something that has not yet been examined in the transitional literature. Using an unbalanced gender cohort is not recommended, given the observations of differences in running mechanics and potential differences in injury risks between males and females [11, 90]. Clearly reporting the injury and dropout rate in both the intervention and control groups is essential, as well as reporting the gender of the injury, for gender difference analysis. Finally, researchers can expect an attrition rate of ~ 16% when planning initial sample sizes.

## Conclusion

Twenty studies have been presented examining the transition to MFW. Whilst the duration and inclusion of exercises and gait retraining was varied, almost all studies implemented a careful progression of exposure to MFW over time. The main outcomes of this transition included limited evidence of benefits of running in MFW for running economy, and muscle development. However, caution is advised with regard to bony injury risk in the initial period, with higher loading rates and plantar pressures observed. Despite the suggested dangers of making this transition, the injury incidence comparing the MFW transition participants to control participants appears equivocal. Finally, several important recommendations for clinical practice and future research have been presented. It is hoped that this paper will present important first steps in unifying the process of transitioning to MFW, both for academic and clinical use.

## References

[1] Running USA (2014). 2014 state of the sport—part II: running industry report.

[2] Kluitenberg B, van Middelkoop M, Diercks R, van der Worp H (2015). What are the differences in injury proportions between different populations of runners? A systematic review and meta-analysis. Sport Med.

[3] Hryvniak D, Dicharry J, Wilder R (2014). Barefoot running survey: evidence from the field. J Sport Heal Sci.

[4] Rothschild CE (2012). Primitive running. J Strength Cond Res.

[5] Gallant JL, Pierrynowski MR (2014). A theoretical perspective on running-related injuries. J Am Podiatr Med Assoc.

[6] Lieberman DE, Venkadesan M, Werbel WA, Daoud AI, D’Andrea S, Davis IS (2010). Foot strike patterns and collision forces in habitually barefoot versus shod runners. Nature.

[7] Warne JP, Kilduff SM, Gregan BC, Nevill AM, Moran KA, Warrington GD (2014). A 4-week instructed minimalist running transition and gait-retraining changes plantar pressure and force. Scand J Med Sci Sports.

[8] Warne JP, Smyth BP, Fagan JO, Hone ME, Richter C, Nevill AM, et al. Kinetic changes during a six-week minimal footwear and gait-retraining intervention in runners. J Sports Sci. 2016:1–9. [cited 2016 Oct 25] Available from: https://www.tandfonline.com/doi/full/10.1080/02640414.2016.1224916. 10.1080/02640414.2016.1224916 27571390

[9] Willson JD, Bjorhus JS, Williams DSB, Butler RJ, Porcari JP, Kernozek TW (2014). Short-term changes in running mechanics and foot strike pattern after introduction to minimalistic footwear. PM R.

[10] Fuller JT, Bellenger CR, Thewlis D, Tsiros MD, Buckley JD (2015). The effect of footwear on running performance and running economy in distance runners. Sport Med.

[11] Johnson AW, Myrer JW, Mitchell UH, Hunter I, Ridge ST (2016). The effects of a transition to minimalist shoe running on intrinsic foot muscle size. Int J Sports Med.

[12] Davis IS (2014). The re-emergence of the minimal running shoe. J Orthop Sport Phys Ther.

[13] Rothschild C (2012). Running barefoot or in minimalist shoes: Evidence or Conjecture?. Strength Cond J.

[14] Lieberman DE (2012). What we can learn about running from barefoot running: An Evolutionary Medical Perspective. Exerc Sport Sci Rev.

[15] Diebal AR, Gregory R, Alitz C, Gerber JP (2012). Forefoot running improves pain and disability associated with chronic exertional compartment syndrome. Am J Sports Med.

[16] Hobara H, Kato E, Kobayashi Y, Ogata T (2012). Sex differences in relationship between passive ankle stiffness and leg stiffness during hopping. J Biomech.

[17] Bramble DM, Lieberman DE (2004). Endurance running and the evolution of Homo. Nature.

[18] Guthold R, Ono T, Strong KL, Chatterji S, Morabia A (2008). Worldwide variability in physical inactivity: a 51-country survey. Am J Prev Med.

[19] Esculier J-F, Dubois B, Dionne CE, Leblond J, Roy J-S, Gent R (2015). A consensus definition and rating scale for minimalist shoes. J Foot Ankle Res.

[20] Moher D, Liberati A, Tetzlaff J, Altman DG, PRISMA Group (2009). Preferred reporting items for systematic reviews and meta-analyses: the PRISMA statement. Ann Intern Med.

[21] Downs SH, Black N (1998). The feasibility of creating a checklist for the assessment of the methodological quality both of randomised and non-randomised studies of health care interventions. J Epidemiol Community Health.

[22] Almeida MO, Davis IS, Lopes AD (2015). Biomechanical differences of foot strike patterns during running: a systematic review with meta-analysis. J Orthop Sports Phys Ther.

[23] Hall JPL, Barton C, Jones PR, Morrissey D (2013). The biomechanical differences between barefoot and shod distance running: a systematic review and preliminary meta-analysis. Sport Med.

[24] Ridge ST, Johnson AW, Mitchell UH, Hunter I, Robinson E, Rich BSE (2013). Foot bone marrow edema after a 10-wk transition to minimalist running shoes. Med Sci Sports Exerc.

[25] Ridge ST, Standifird T, Rivera J, Johnson AW, Mitchell U, Hunter I (2015). The effect of training in minimalist running shoes on running economy. J Sports Sci Med.

[26] Campitelli NA, Spencer SA, Bernhard K, Heard K, Kidon A (2016). Effect of Vibram FiveFingers minimalist shoes on the abductor hallucis muscle. J Am Podiatr Med Assoc.

[27] Chen TL-W, Sze LKY, Davis IS, RTH C (2016). Effects of training in minimalist shoes on the intrinsic and extrinsic foot muscle volume. Clin Biomech.

[28] McCarthy C, Fleming N, Donne B, Blanksby B. 12 weeks of simulated barefoot running changes foot-strike patterns in female runners. Int J Sports Med. 2014; 35:443–4450. [cited 2016 Jun 23] Available from: http://www.ncbi.nlm.nih.gov/pubmed/24048910. 10.1055/s-0033-1353215 24048910

[29] Miller EE, Whitcome KK, Norton HL, Dyer RE (2014). The effect of minimal shoes on arch structure and intrinsic foot muscle strength. J Sport Heal Sci.

[30] Dubois B, Esculier J-F, Frémont P, Moore L, Richards C (2015). Effects of minimalist and traditional running shoes on injury rates: a pilot randomised controlled trial. Footwear Sci.

[31] Ryan M, Elashi M, Newsham-West R, Taunton J (2014). Examining injury risk and pain perception in runners using minimalist footwear. Br J Sports Med.

[32] Warne JP, Moran KA, Warrington GD (2015). Eight weeks gait retraining in minimalist footwear has no effect on running economy. Hum Mov Sci.

[33] Fuller JT, Thewlis D, Buckley JD, Brown NAT, Hamill J, Tsiros MD. Body mass and weekly training distance influence the pain and injuries experienced by runners using minimalist shoes. Am J Sports Med. 2017;45(5):1162–1170. [cited 2017 Feb 20] Available from: http://www.ncbi.nlm.nih.gov/pubmed/28129518. 10.1177/0363546516682497 28129518

[34] Bellar D, Judge LW (2015). Effect of training in minimalist footwear on oxygen consumption during walking and running. Biol Sport.

[35] Khowailed IA, Petrofsky J, Lohman E, Daher N (2015). Six weeks habituation of simulated barefoot running induces neuromuscular adaptations and changes in foot strike patterns in female runners. Med Sci Monit.

[36] Moore IS, Pitt W, Nunns M, Dixon S (2014). Effects of a seven-week minimalist footwear transition programme on footstrike modality, pressure variables and loading rates. Footwear Sci.

[37] Joseph MF, Histen K, Arntsen J, L’hereux L, Defeo C, Lockwood D, et al. Achilles tendon adaptation during transition to a minimalist running style. J Sport Rehabil. 2016:1–17. [cited 2017 Feb 20] Available from: http://journals.humankinetics.com/doi/10.1123/jsr.2016-0007. 10.1123/jsr.2016-0007 27632879

[38] Warne JP, Warrington GD (2014). Four-week habituation to simulated barefoot running improves running economy when compared with shod running. Scand J Med Sci Sports.

[39] Azevedo AP d S, Nóbrega C, Amadio AC, Serrão JC, Azevedo AP d S, Nóbrega C (2016). Adherence to six months of instructed minimalist and barefoot running training. Rev Bras Med do Esporte.

[40] Dubois B, Esculier J-F, Frémont P, Moore L, Richards C. Effects of minimalist and traditional running shoes on injury rates: a pilot randomised controlled trial. 10.1080/19424280.2015.1049300. Taylor & Francis; 2015.

[41] Bellar D, Judge LW (2015). Effect of training in minimalist footwear on oxygen consumption during walking and running. Biol Sport.

[42] Giandolini M, Horvais N, Farges Y, Samozino P, Morin JB (2013). Impact reduction through long-term intervention in recreational runners: Midfoot strike pattern versus low-drop/low-heel height footwear. Eur J Appl Physiol.

[43] Sale DG (1988). Neural adaptation to resistance training. Med Sci Sports Exerc.

[44] Hart PM, Smith DR. Preventing running injuries through barefoot activity. JOPERD J Phys Educ Recreat Danc. 2008:50–3. Available from: http://www.tandfonline.com/doi/abs/10.1080/07303084.2008.10598165.

[45] Robbins S, Gouw GJ, McClaran J, Waked E (1993). Protective sensation of the plantar aspect of the foot. Foot Ankle.

[46] Rothschild C (2012). Running barefoot or in minimalist shoes. Strength Cond J.

[47] Buist I, Bredeweg SW, van Mechelen W, Lemmink KAPM, Pepping G-J, Diercks RL (2008). No effect of a graded training program on the number of running-related injuries in novice runners: a randomized controlled trial. Am J Sports Med.

[48] Oestergaard Nielsen R, Buist I, Sørensen H, Lind M, Rasmussen S (2012). Training errors and running related injuries: a systematic review. Int J Sports Phys Ther.

[49] Van Gent RN, Siem D, Van Middeloop M, Van Os AG, Bierma-Zeinstra SMA, Koes BW (2007). Incidence and determinants of lower extremity running injuries in long distance runners: a systematic review. Sport en Geneeskd.

[50] Salzler MJ, Bluman EM, Noonan S, Chiodo CP, de Asla RJ (2012). Injuries observed in minimalist runners. Foot Ankle Int.

[51] Salzler MJ, Kirwan HJ, Scarborough DM, Walker JT, Guarino AJ, Berkson EM (2016). Injuries observed in a prospective transition from traditional to minimalist footwear: correlation of high impact transient forces and lower injury severity. Phys Sportsmed.

[52] Divert C, Baur H, Mornieux G, Mayer F, Belli A (2005). Stiffness adaptations in shod running. J Appl Biomech.

[53] Malisoux L, Ramesh J, Mann R, Seil R, Urhausen A, Theisen D (2015). Can parallel use of different running shoes decrease running-related injury risk?. Scand J Med Sci Sport.

[54] Paquette MR, Milner CE, Melcher DA. Foot contact angle variability during a prolonged run with relation to injury history and habitual foot strike pattern. Scand J Med Sci Sports. 2016; [cited 2016 Oct 25];n/a-n/a. Available from: http://doi.wiley.com/10.1111/sms.12647. 10.1111/sms.12647 26804467

[55] Barrett R, Noordegraaf MV, Morrison S (2008). Gender differences in the variability of lower extremity kinematics during treadmill locomotion. J Mot Behav.

[56] Hamill J, van Emmerick R, Heiderscheit B, Li L (1999). A dynamic systems approach to lower extremity running injuries. Clin Biomech.

[57] Heiderscheit B, Hamill J, van Emmerik R (2002). Variability of stride characteristics and joint coordination among individuals with unilateral patellofemoral pain. J Appl Biomech.

[58] Dufek JS, Mercer J a, Teramoto K, Mangus BC, Freedman J a (2008). Impact attenuation and variability during running in females: a lifespan investigation. J Sport Rehabil.

[59] van Mechelen W (1992). Running injuries. A review of the epidemiological literature. Sports Med.

[60] Hamill J, Palmer C, Van Emmerik REA. Coordinative variability and overuse injury. Sports Med Arthrosc Rehabil Ther Technol. 2012;4:–45. [cited 2016 Oct 25] Available from: http://www.ncbi.nlm.nih.gov/pubmed/23186012. 10.1186/1758-2555-4-45 PMC3536567 23186012

[61] Hamill J, van Emmerik REA, Heiderscheit BC, Li L (1999). A dynamical systems approach to lower extremity running injuries. Clin Biomech.

[62] Jung D-Y, Kim M-H, Koh E-K, Kwon O-Y, Cynn H-S, Lee W-H (2011). A comparison in the muscle activity of the abductor hallucis and the medial longitudinal arch angle during toe curl and short foot exercises. Phys Ther Sport.

[63] Olsen O-E, Myklebust G, Engebretsen L, Holme I, Bahr R (2005). Exercises to prevent lower limb injuries in youth sports: cluster randomised controlled trial. BMJ.

[64] Tenforde AS, Sayres LC, McCurdy ML, Collado H, Sainani KL, Fredericson M (2011). Overuse injuries in high school runners: lifetime prevalence and prevention strategies. PM R.

[65] Johnston CAM, Taunton JE, Lloyd-Smith DR, DC MK (2003). Preventing running injuries. Practical approach for family doctors. Can Fam Physician.

[66] Williams DS, McClay IS, Manal KT. Lower extremity mechanics in runners with a converted forefoot strike pattern. J Appl Biomech. 2000:210–8.

[67] Crowell HP, Davis IS (2011). Gait retraining to reduce lower extremity loading in runners. Clin Biomech (Bristol, Avon).

[68] Shih Y, Lin KL, Shiang TY (2013). Is the foot striking pattern more important than barefoot or shod conditions in running?. Gait Posture.

[69] De Wit B, De Clercq D, Aerts P (2000). Biomechanical analysis of the stance phase during barefoot and shod running. J Biomech.

[70] Kulmala JP, Avela J, Pasanen K, Parkkari J (2013). Forefoot strikers exhibit lower running-induced knee loading than rearfoot strikers. Med Sci Sports Exerc.

[71] Diebal a R, Gregory R, Alitz C, Gerber JP (2012). Forefoot running improves pain and disability associated with chronic exertional compartment syndrome. Am J Sports Med.

[72] Roper JL, Dufek JS, Mermier CM (2014). Gait retraining with foot strike patterns as management for patellofemoral pain syndrome: a brief review. Int J Sport Sci.

[73] Hobara H, Sato T, Sakaguchi M, Sato T, Nakazawa K (2012). Step frequency and lower extremity loading during running. Int J Sports Med.

[74] Lenhart RL, Thelen DG, Wille CM, Chumanov ES, Heiderscheit BC (2014). Increasing running step rate reduces patellofemoral joint forces. Med Sci Sports Exerc.

[75] Heiderscheit BC, Chumanov ES, Michalski MP, Wille CM, Ryan MB (2011). Effects of step rate manipulation on joint mechanics during running. Med Sci Sports Exerc.

[76] Goss DL, Gross MT. A review of mechanics and injury trends among various running styles. US Army Med Dep J. 2012:62–71. Available from: http://www.ncbi.nlm.nih.gov/pubmed/22815167. 22815167

[77] Arendse RE, Noakes TD, Azevedo LB, Romanov N, Schwellnus MP, Fletcher G (2004). Reduced eccentric loading of the knee with the pose running method. Med Sci Sports Exerc.

[78] Dallam GM, Wilber RL, Jadelis K, Fletcher G, Romanov N (2005). Effect of a global alteration of running technique on kinematics and economy. J Sports Sci.

[79] Napier C, Cochrane CK, Taunton JE, Hunt MA (2015). Gait modifications to change lower extremity gait biomechanics in runners: a systematic review. Br J Sports Med.

[80] Wrisberg CA. Sport skill instruction for coaches. Human Kinetics. 2007;9:127–36. ISBN-13:9780736039871.

[81] Noehren B, Scholz J, Davis I (2011). The effect of real-time gait retraining on hip kinematics, pain and function in subjects with patellofemoral pain syndrome. Br J Sports Med.

[82] Willy RW, Scholz JP, Davis IS (2012). Mirror gait retraining for the treatment of patellofemoral pain in female runners. Clin Biomech.

[83] Fletcher JR, Esau SP, Macintosh BR (2009). Economy of running: beyond the measurement of oxygen uptake. J Appl Physiol.

[84] Gruber AH, Umberger BR, Braun B, Hamill J (2013). Economy and rate of carbohydrate oxidation during running with rearfoot and forefoot strike patterns. J Appl Physiol.

[85] Cavanagh PR, Williams KR (1982). The effect of stride length variation on oxygen uptake during distance running. Med Sci Sports Exerc.

[86] Tseh W, Caputo JL, Morgan DW (2008). Influence of gait manipulation on running economy in female distance runners. J Sport Sci Med.

[87] Cauthon DJ, Langer P, Coniglione TC (2013). Minimalist shoe injuries: three case reports. Foot (Edinb).

[88] Giuliani J, Masini B, Alitz C, Owens LBD (2011). Barefoot-simulating footwear associated with metatarsal stress injury in 2 runners. Orthopedics.

[89] Altman AR, Davis IS. Prospective comparison of running injuries between shod and barefoot runners. Br J Sports Med. 2015:1–6. Available from: http://www.ncbi.nlm.nih.gov/pubmed/26130697. 10.1136/bjsports-2014-094482 26130697

[90] Ferber R, Davis IM, Williams DS (2003). Gender differences in lower extremity mechanics during running. Clin Biomech.

[91] Davis IS (2014). The re-emergence of the minimal running shoe. J Orthop Sports Phys Ther.

[92] Zadpoor AA, Nikooyan AA (2011). The relationship between lower-extremity stress fractures and the ground reaction force: a systematic review. Clin Biomech.

[93] Tonoli DC, Cumps E, Aerts I, Verhagen E, Meeusen R. Incidence, risk factors and prevention of running related injuries in long-distance running: a systematic review. Sport Geneeskd. 2010:12–8.

[94] Yeung EW, Yeung SS (2001). A systematic review of interventions to prevent lower limb soft tissue running injuries. Br J Sport Med.

[95] Chuter V, Janse de Jonge XAK (2012). Proximal and distal contributions to lower extremity injury: a review of the literature. Gait Posture.

[96] Murphy DF, Connolly DJ, Beynnon BD (2003). Risk factors for lower extremity injury: a review of the literature. Br J Sports Med.

[97] van der Worp MP, ten Haaf DSM, van Cingel R, de Wijer A, Nijhuis-van der Sanden MWG, Staal JB (2015). Injuries in runners; a systematic review on risk factors and sex differences. PLoS One.

[98] Hulme A, Nielsen RO, Timpka T, Verhagen E, Finch C. Risk and protective factors for middle- and long-distance running-related injury. Sport Med. 2016:1–18. [cited 2016 Nov 1] Available from: http://link.springer.com/10.1007/s40279-016-0636-4. 10.1007/s40279-016-0636-4 27785775

[99] Braunstein B, Arampatzis A, Eysel P, Brüggemann GP (2010). Footwear affects the gearing at the ankle and knee joints during running. J Biomech.

[100] Almonroeder T, Willson JD, Kernozek TW (2013). The effect of foot strike pattern on Achilles tendon load during running. Ann Biomed Eng.

[101] Bonacci J, Saunders PU, Hicks A, Rantalainen T, Vicenzino BGT, Spratford W (2013). Running in a minimalist and lightweight shoe is not the same as running barefoot: a biomechanical study. Br J Sports Med.

